# Self‐reported and actual adherence to the Tokyo guidelines in the European snapshot audit of complicated calculous biliary disease

**DOI:** 10.1002/bjs5.50294

**Published:** 2020-05-17

**Authors:** G. A. Bass, A. E. Gillis, Y. Cao, S. Mohseni, European Society for Trauma and Emergency Surgery (ESTES) Cohort Studies Group

**Affiliations:** ^1^ Department of Surgery Tallaght University Hospital Dublin Ireland; ^2^ Departments of Clinical Epidemiology and Biostatistics Örebro Sweden; ^3^ Surgery Örebro University School of Medical Sciences Örebro Sweden

## Abstract

**Background:**

Complicated acute biliary calculous disease poses clinical challenges. The European Society of Trauma and Emergency Surgery (ESTES) snapshot audit of complicated biliary calculous disease aims to make novel comparisons between self‐reported institutional adherence to the Tokyo guidelines (TG18) and ‘real‐world’ contemporary practice across Europe.

**Methods:**

A preplanned analysis of a prospective observational multicentre audit that captured patients undergoing emergency admission for complicated biliary calculous disease (complicated cholecystitis, biliary pancreatitis, or choledocholithiasis with or without cholangitis) between 1 and 31 October 2018 was performed. An anonymized survey was administered to participating sites.

**Results:**

Following an open call for participation, 25 centres from nine countries enrolled 338 patients. All centres completed the anonymized survey. Fifteen centres (60 per cent) self‐reported that a minority of patients were treated surgically on index admission, favouring interval cholecystectomy. This was replicated in the snapshot audit, in which 152 of 338 patients (45·0 per cent) underwent index admission cholecystectomy, 17 (5·0 per cent) had interval cholecystectomy, and the remaining 169 (50·0 per cent) had not undergone surgery by the end of the 60‐day follow‐up. Centres that employed a dedicated acute care surgery model of care were more likely to perform index admission cholecystectomy compared with a traditional general surgery ‘on call’ service (57 *versus* 38 per cent respectively; odds ratio 2·14 (95 per cent c.i. 1·37 to 3·35), *P* < 0·001). Six centres (24 per cent) self‐reported routinely performing blood cultures in acute cholecystitis; patient‐level audit data revealed that blood cultures were done in 47 of 154 patients (30·5 per cent). No centre self‐reported omitting antibiotics in the management of acute cholecystitis, and 144 of 154 (93·5 per cent) of patients in the snapshot audit received antibiotics during their index admission.

**Conclusion:**

Awareness of TG18 recommendations was high, but self‐reported adherence and objective snapshot audit data showed low compliance with TG18 in patients with complicated acute biliary calculous disease.

## Graphical abstract

The Tokyo Guidelines 2018 (TG18) describe expert consensus for best practice in the management of complex acute biliary calculous disease. Self‐reported adherence (and limitations to this) were compared with ‘real‐world’ patient‐level data from a prospective observational study, the ESTES snapshot audit. Recognition of TG18 was high, but adherence to guidance on both index admission cholecystectomy and microbiological culture‐guided antibiotic therapy was poor.

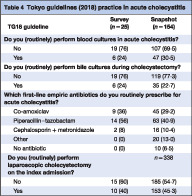

Low self‐reported and actual adherence to guidelines

## Abstract (es)

**Antecedentes:**

La complicación aguda de la litiasis biliar (*complicated acute biliary calculous disease,* CABCD) plantea retos clínicos. Esta auditoría de la Sociedad Europea de Trauma y Cirugía de Urgencias (*European Society of Trauma and Emergency Surgery*, ESTES) de la CABCD tuvo como objetivo comparar el conocimiento teórico de las recomendaciones de Tokio (TG18) y la “práctica real” en Europa.

**Métodos:**

Se efectuó un análisis pre‐establecido de los datos de una auditoría prospectiva, observacional y multicéntrica que incluyó los pacientes ingresados de urgencia por CABCD (es decir, colecistitis complicada, pancreatitis biliar o coledocolitiasis con o sin colangitis) entre el 1 y el 31 de octubre de 2018. Además, se realizó una encuesta anónima en los centros participantes.

**Resultados:**

Tras una convocatoria abierta, 25 centros de 9 países incluyeron 338 pacientes. Todos los centros completaron la encuesta anónima. El 60% de los centros reconocieron que trataban en el mismo ingreso una minoría de los pacientes y que favorecían la colecistectomía diferida. Ello se reprodujo en la auditoria, donde a 152/338 (44,9%) de los pacientes se realizó la colecistectomía en el mismo ingreso, a 17/338 (5%) se realizó una colecistectomía diferida y que a 169/338 (50%) todavía no se había realizado ninguna intervención en los 60 días de seguimiento. Los centros que seguían el Modelo de Atención Quirúrgica Urgente tenían mayores probabilidades de realizar la colecistectomía en el mismo ingreso en comparación con un servicio de cirugía general tradicional 'de guardia' (57% *versus* 38,4%, razón de oportunidades, *odds ratio*, OR 2,14 (i.c. del 95% 1,37‐3,35), *P* < 0,001)). El 24% de los centros afirmaron realizar hemocultivos de rutina en la colecistitis aguda. Sin embargo, los datos de la auditoría revelaron que solamente 47/154 (30,5%) de los pacientes tenían hemocultivos. Ningún centro declaró no administrar antibióticos en el tratamiento de la colecistitis aguda, mientras que 144/154 (93,5%) de los pacientes de la auditoría no recibieron antibióticos durante el ingreso.

**Conclusiones:**

El conocimiento de las recomendaciones de TG18 fue alto. Sin embargo, la observancia reconocida por los centros y los datos objetivos de la auditoría muestran que el cumplimiento en los pacientes con CABCD es bajo.

## Introduction

Acute complications of biliary calculi, such as complicated cholecystitis, choledocholithiasis with or without cholangitis, and biliary pancreatitis, commonly require urgent hospital admission for surgical care^1^, ^2^. These conditions are morbid and complex to manage^1^, ^3^, ^4^, ^5^, ^6^. Despite the frequency of presentation of these patients, clinical equipoise remains around the optimal timing and mode of therapy^7^, ^8^, ^9^, ^10^.

Efforts have been made to achieve expert consensus on the diagnosis and management of disease conditions caused by biliary calculi, in the form of the Tokyo guidelines, which were updated most recently in 2018 (TG18)^2^, ^11^, ^12^, ^13^. The TG18 expert group^2^ proposed a management bundle emphasizing the prompt diagnosis of acute cholecystitis, biliary pancreatitis and cholangitic choledocholithiasis, and early evaluation of surgical risk, with the commencement of appropriate resuscitation and antimicrobial administration. To guide appropriate antimicrobial management based on local antimicrobial susceptibility data, blood and/or bile cultures are strongly recommended in TG18^14^. For definitive treatment of moderate or severe disease presentation, TG18 advocates early cholecystectomy (within 7 days, but preferably within 72 h) and/or biliary drainage where patient factors allow, reserving delayed cholecystectomy for cases in which delayed presentation, negative physiological factors or patient co‐morbidity favoured non‐operative management^2^, ^11^, ^12^. Recognizing there was no absolute standard definition of interval cholecystectomy (which has been defined in various different ways, including ‘after diagnosis’ or ‘after the symptoms diminished’), TG18 adopted the most common definition as elective readmission for operative management ‘after at least 6 weeks’^15^. Where local surgical expertise or critical care capacity is not available, TG18 urges outbound transfer of these patients to a higher level of care^2^.

The present authors hypothesized that, although regional and patient heterogeneity may account for some of the variability that could be expected in different clinical practices to treat these conditions, other causes such as unit policies and individual surgeon preference might also influence the treatment decisions^4^, ^13^. In this study, 25 different hospitals across Europe and North America were studied to investigate whether heterogeneity in practice and divergence from TG18 guidance was reflective of guideline awareness, individual surgeon preference, differences in models of acute surgical care delivery, or simply the exigencies of real‐world limitations on practice.

## Methods

A prospective observational multicentre audit was conducted in line with a prespecified protocol that was registered with ClinicalTrials.gov (trial number NCT03610308). The audit enrolled all consecutive patients admitted with complicated biliary calculous disease during the month of October 2018, and followed these patients for 60 days after admission (up to 31 December 2018). The database was closed for analysis on 1 February 2019. In May 2019, an anonymized follow‐up survey was completed by all 25 centres, assessing self‐reported awareness of and adherence to recommendations outlined in TG18. Survey respondents were asked to classify the model of unscheduled surgical care employed at their surgical department or hospital into one of the following categories: a dedicated acute care/emergency surgery service line (separate from elective surgical care) or a traditional ‘on call’ emergency service provided by general surgeons (such as upper gastrointestinal, breast, hepatobiliary or colorectal surgeons) with a primary commitment to elective surgical care.

### Patient eligibility

All adult patients (aged 18 years or over) admitted for acute calculous cholecystitis (American Association for the Surgery of Trauma (AAST) severity grade I–II or above), choledocholithiasis or complications of cholelithiasis and/or choledocholithiasis, or biliary pancreatitis were included in the study. Surgical procedures performed on these patients included cholecystectomy (open, laparoscopic or laparoscopic converted to open), choledochotomy/common bile duct (CBD) exploration (open or laparoscopic) or pancreatic necrosectomy. Data on endoscopic retrograde cholangiopancreatography (ERCP), radiological percutaneous cholecystostomy (transhepatic or transperitoneal), percutaneous transhepatic drainage, stone removal or stent placement were also collected. Patients with uncomplicated biliary colic or biliary dyskinesia were excluded from the study.

### Ethical considerations

All participating centres had institutional review board approval or equivalent. No patient consent was sought as the study was purely observational and did not change the medical course of any patient. All data were deidentified at source when uploaded to the study database.

### Data capture

Data were recorded contemporaneously and stored on a secure user‐encrypted online platform (REDCap®; https://www.project‐redcap.org/) without patient‐identifiable information. Centres were asked to validate that all eligible patients during the study period had been entered, and to attain more than 95 per cent completeness of data field entry before final submission. Quality assurance mentorship was provided by at least one consultant or attending‐level surgeon at every participating site.

### Outcome measures

The primary outcome measures were self‐reported *versus* actual adherence to TG18 recommendations concerning the timing of operative management and antimicrobial therapy. The secondary outcome measure was to identify variation in the use of index admission cholecystectomy, stratified by surgical specialty and hospital type.

### Statistical analysis

Descriptive and inferential statistical analyses were performed using Stata® 15.1 (StataCorp, College Station, Texas, USA) and the jamovi project version 1.2.16 (www.jamovi.com; 2019) using R 3.6.0 El Capitan (The R Foundation for Statistical Computing, Vienna, Austria). Measures of central tendency are presented as mean(s.d.) (range) or median (i.q.r.) values, and comparisons were made with the χ^2^ test or ANOVA, as appropriate. An α significance level of 0·05 was used throughout.

## Results

Following an open call for participation by the European Society for Trauma and Emergency Surgery (ESTES) in May 2018, 25 centres from nine countries (Austria, Ireland, Italy, Portugal, Romania, Spain, Sweden, UK, USA) completed the local ethical approval process and proceeded to enrol patients prospectively. Fourteen (56 per cent) of the 25 centres described themselves as a university hospital or tertiary referral centre, and the remaining 11 (44 per cent) described themselves as a general/community hospital. The median catchment population of centres was 500 000 people. The majority of centres reported high volumes of eligible patients, with 22 (88 per cent) of the 25 centres performing more than 100 elective laparoscopic cholecystectomies per year. Similarly, 24 centres (96 per cent) reported receiving more than 300 admissions per annum for symptomatic biliary calculous disease.

### Model of unscheduled surgical care

A dedicated acute care/emergency surgery service line (separate from that for elective general surgery) existed in seven (28 per cent) of the 25 centres, whereas general surgery on‐call managed and operated on patients in 18 (72 per cent) of the centres. Previous training in hepatopancreatobiliary (HPB) surgery had been undertaken by surgeons in eight (32 per cent) of the 25 centres (*Table* *1*).

**Table 1:** Demographic characteristics of centres

	No. of centres (*n* = 25)
**Who provides unscheduled surgical care?**	
General surgery on‐call	18
Emergency/acute care surgery	7
**Have you undertaken training in hepatobiliary surgery?**	
No	17
Yes	8

### Patient demographics

A total of 338 consecutive patients admitted between 1 and 31 October 2018 were enrolled in the study and followed up for 60 days after admission (latest patient to 31 December 2018). Women outnumbered men (53·8 *versus* 46·2 per cent). The mean(s.d.) age of patients at the time of diagnosis was 64·5(18·4) years. The mean(s.d.) BMI was calculated as 28·5(6·4) kg/m^2^. Some 16·3 per cent of the patients were current smokers of tobacco products, 34·9 per cent were ex‐smokers (stopped smoking more than 6 weeks before admission), and 48·8 per cent had never smoked. The mean(s.d.) age‐adjusted Charlson Co‐morbidity Index score was 6·26(4·1), and the mean(s.d.) APACHE‐II score was 12·3(7·6) (*Table* *2*).

**Table 2:** Demographic characteristics of patients

	No. of patients* (*n* = 338)
**Age (years)** **†**	64·5(18·4)
**Sex ratio (M** : **F)**	156 : 182
**BMI (kg/m** ^**2**^ **)** **†**	28·5(6·4)
**Smoking status**	
Smoker	55 (16·3)
Non‐smoker	165 (48·8)
Ex‐smoker	118 (34·9)
**ASA fitness grade**	*n* = 333
I	62 (18·6)
II	149 (44·7)
III	95 (28·5)
IV	27 (8·1)
**Age‐adjusted CCI score** **†**	6·26(4·1)
**APACHE‐II score** **†**	12·3(7·6)
**Diagnosis on admission**	
Cholecystitis	154 (45·6)
Biliary pancreatitis	71 (21·0)
Choledocholithiasis with cholangitis	47 (13·9)
Choledocholithiasis without cholangitis	61 (18·0)
Bilioenteric fistula	5 (1·5)

* With percentages in parentheses unless indicated otherwise;

† values are mean(s.d.). CCI, Charlson Co‐morbidity Index; APACHE, Acute Physiology And Chronic Health Evaluation.

### Diagnosis

Acute calculous cholecystitis was present in 154 (45·6 per cent) of the 338 patients, acute biliary pancreatitis in 71 (210 per cent) and choledocholithiasis in 108 (32·0 per cent), of whom 47 (43·5 per cent) had cholangitis. Five patients (1·5 per cent) were admitted for treatment of Mirizzi syndrome or bilioenteric fistula (*Table* *2*).

### Self‐reported adherence to Tokyo guidelines 2018 *versus* audited practice

The Tokyo guidelines (TG18) set out a number of best‐practice parameters deemed by expert consensus to contribute to optimal care. Self‐reported (retrospective) adherence to the guidelines was assessed, along with prospective data on adherence from the ESTES snapshot audit of the same centres.

#### Cholecystectomy at index admission

Survey respondents were asked to estimate the percentage of patients in their practice who underwent cholecystectomy during the index hospital stay. Fifteen (60 per cent) of the 25 centres self‐reported that a minority of patients with acute cholecystitis had cholecystectomy on the index admission, favouring interval cholecystectomy. When polled on reasons for this practice (multiple answers were permitted), acute care surgeons solely cited lack of emergency operating room access, while the responses from general surgery centres were more diverse (*Table* *3*). Centres using an acute care/emergency surgery model of care self‐reported performing laparoscopic cholecystectomy during the index admission in a mean(s.d.) of 78·0(9·4) per cent of patients, compared with 42·7(8·3) per cent of patients in centres with a general surgeon on‐call (*P* < 0·001).

**Table 3:** Comparison of index admission laparoscopic cholecystectomy practice and attitudes by care model: acute care surgery *versus* general surgery on call

TG18 guideline	Acute care surgery centres (*n* = 7)	General surgery centres (*n* = 18)	*P* *
**Index admission laparoscopic cholecystectomy (snapshot audit)**			
Yes	72 of 126 (57·1)	81 of 211 (38·4)	
**When you choose interval over index admission laparoscopic cholecystectomy, what are the main reasons?**			
Delay in presentation or diagnosis	0 (0)	3 (17)	0·259
Access to operating room	1 (14)	7 (39)	0·246
Surgeon preference	0 (0)	10 (56)	0·013
Patient preference	0 (0)	0 (0)	

Values in parentheses are percentages.

* χ^2^ test.

When snapshot audit data were analysed, centres employing a dedicated acute care surgery model of care were significantly more likely to perform cholecystectomy on the index admission than those with a traditional on‐call service provided by general surgeons primarily committed to elective care (57·1 *versus* 38·4 per cent respectively; OR 2·14 (95 per cent c.i. 1·37 to 3·35), *P* < 0·001) (*Table* *3*).

Previous training in HPB surgery did not significantly influence the declared *a priori* decision to perform laparoscopic cholecystectomy on index admission: (51·5 per cent for surgeons without HPB training *versus* 47·9 per cent for those with HPB training; *P* = 0·508).

Of the 338 patients enrolled in the study, 169 (50·0 per cent) had a surgical intervention and 169 had not undergone surgery by the end of the 60‐day follow‐up period. Of patients who had a cholecystectomy, 152 (89·9 per cent) had the operation during the index admission, and only 17 (10·1 per cent) were reported as having been operated on after discharge from the index admission but before closure of the study database. The median interval from index admission to cholecystectomy was 66 (i.q.r. 43–71) days.

#### Blood and bile cultures, and antimicrobial strategy in acute cholecystitis

Survey respondents were asked whether, in their practice, admission blood and intraoperative bile cultures were performed for patients admitted with acute cholecystitis. Six (24 per cent) of the 25 centres stated that they routinely performed blood cultures, and six (24 per cent) reported routinely performing intraoperative bile cultures during cholecystectomy. When patient‐level data from the snapshot audit of the same centres were reviewed, 47 (30·5 per cent) of 154 patients with acute cholecystitis had blood cultures drawn, and 35 (22·7 per cent) had intraoperative bile cultures sent for microbiological analysis (*Table* *4*). Reported practice did not differ significantly from observed practice (*P* = 0·944).

**Table 4:** Tokyo guidelines (2018) practice in acute cholecystitis

TG18 guideline	Survey (*n* = 25)	Snapshot (*n* = 154)
**Do you (routinely) perform blood cultures in acute cholecystitis?**		
No	19 (76)	107 (69·5)
Yes	6 (24)	47 (30·5)
**Do you (routinely) perform bile cultures during cholecystectomy?**		
No	19 (76)	119 (77·3)
Yes	6 (24)	35 (22·7)
**Which first‐line empirical antibiotics do you routinely prescribe for acute cholecystitis?**		
Co‐amoxiclav	9 (36)	45 (29·2)
Piperacillin–tazobactam	14 (56)	63 (40·9)
Cephalosporin + metronidazole	2 (8)	16 (10·4)
Other	0 (0)	20 (13·0)
No antibiotic	0 (0)	10 (6·5)
**Do you (routinely) perform laparoscopic cholecystectomy on the index admission?**		*n* = 338
No	15 (60)	185 (54·7)
Yes	10 (40)	153 (45·3)

Values in parentheses are percentages.

#### Blood cultures and antimicrobial strategy in acute pancreatitis

Survey respondents were also asked whether, in their practice, admission blood cultures were performed for patients admitted with acute biliary pancreatitis. Eight (32 per cent) of the 25 centres stated that they routinely performed blood cultures on admission. When patient‐level data from the snapshot audit of the same centres were reviewed, 28 per cent (20 of 71) of patients with acute pancreatitis had blood cultures drawn for microbiological analysis (*Table* *5*). Reported practice did not differ significantly from observed practice (*P* = 0·717). Eleven centres (44 per cent) elected not to commence antibiotics in patients with biliary pancreatitis. Of the 14 centres that did commence empirical antibiotics, piperacillin–tazobactam was the favoured drug (7 of 14, 50 per cent), with meropenem and co‐amoxiclav each favoured in three centres, and cephalosporin plus metronidazole combination in one centre.

**Table 5:** Tokyo guidelines (2018) practice in acute pancreatitis

TG18 guideline	Survey (*n* = 25)	Snapshot (*n* = 71)
**Do you routinely perform blood cultures in biliary pancreatitis?**		
No	17 (68)	51 (72)
Yes	8 (32)	20 (28)
**Which first‐line empirical antibiotics do you prescribe routinely for biliary pancreatitis?**		
Co‐amoxiclav	3 (12)	
Piperacillin–tazobactam	7 (28)	
Cephalosporin + metronidazole	1 (4)	
Meropenem	3 (12)	10 (14)
No antibiotic	11 (44)	

Values in parentheses are percentages.

#### Common bile duct clearance

Addressing the question of the therapeutic sequence for the management of choledocholithiasis in patients fit for cholecystectomy, 20 (80 per cent) of the 25 centres reported that they favoured a staged approach, with upfront ERCP followed by cholecystectomy (either during the same admission or, more commonly, at an interval). A minority of survey respondents favoured simultaneous cholecystectomy and either operative CBD exploration (4 of 25, 16 per cent) or rendezvous intraoperative ERCP (5 of 25, 20 per cent) as a one‐stage procedure (*Table* *6*).

**Table 6:** Common bile duct clearance: surgical, endoscopic and hybrid practices

	No. of centres (*n* = 25)
**Do you perform preoperative ERCP before LC?**	
No	5
Yes	20
**Do you perform LC and CBD clearance?**	
No	21
Yes	4
**Do you perform simultaneous LC and intraoperative rendezvous ERCP?**	
No	20
Yes	5

ERCP, endoscopic retrograde cholangiopancreatography; LC, laparoscopic cholecystectomy; CBD, common bile duct.

## Discussion

Incremental improvements in patient outcomes for certain common surgical conditions are achievable by the standardization of patient care through practice management guidelines^4^, ^16^, ^17^, ^18^. In complex conditions, small cumulative relative risk reductions may be attached to early diagnosis, risk stratification, appropriate resuscitation and directed antimicrobial therapy, as well as prompt surgical, endoscopic or percutaneous radiological intervention^4^, ^17^. Clinical practice guidelines are ‘systematically‐developed statements designed to assist practitioner decisions about appropriate healthcare interventions for specific clinical circumstances’^19^. Of course, no guideline is perfect, neither are guidelines intended to supplant clinical experience or the individual factors contributing to a particular patient's overall condition. Over recent years, however, concerted efforts have been made to aggregate these marginal gains into meaningful outcomes improvements through evidence‐based guidelines for the management of complicated cholecystitis, choledocholithiasis with or without cholangitis, and biliary pancreatitis (TG18)^2^, ^11^, ^12^, ^13^.

The Tokyo guideline TG18 has been promulgated widely, and its apparent success may be measured by how it is now part of the vernacular whenever the management of complicated calculous biliary disease is being discussed^16^. Continuing evidence of medical practice variations and gaps in the quality of care has spurred the rapid development of practice guidelines in most areas of clinical practice. Guidelines have had variable effect on changing physician behaviour, however, and a number of studies^16^, ^19^, ^20^, ^21^, ^22^ have examined clinician adherence to these guidelines as process measures of quality of care. This literature may be compromised by an overreliance on self‐report measures of guideline adherence, because of possible response biases in self‐reports^4^, ^16^, ^17^, ^18^, ^20^. Furthermore, several inherent barriers have been shown to exist in guideline adherence, namely lack of familiarity, agreement, self‐efficacy and external inhibiting factors, outcome expectancy, and the inertia of previous practice^19^.

In the present study, self‐report measures of TG18 compliance among surgical services providing acute surgical care in 25 hospitals across nine countries were compared with real‐world prospective data by snapshot audit from the same centres. The survey asked respondents to report their practice in various domains of TG18, without explicitly referencing these guidelines.

TG18 advocates definitive surgical intervention (usually laparoscopic cholecystectomy) on the index admission in patients with acute cholecystitis or biliary pancreatitis, citing both prospective longitudinal studies and retrospective observational studies that demonstrated a significantly lower incidence of disease recurrence, hospital readmission, and overall disease‐specific complications when the procedure was performed within 7 days of the onset of symptoms in the appropriate patient (recommending within 72 h as preferable)^5^, ^17^, ^23^, ^24^, ^25^, ^26^, ^27^, ^28^, ^29^, ^30^. However, 15 (60 per cent) of the 25 centres in the present study estimated that less than half of their patients routinely had index admission cholecystectomy. Indeed, this estimation was borne out by prospective snapshot audit data from the same centres, in which just 169 (50·0 per cent) of 338 patients underwent cholecystectomy during the study period, of whom 152 (89·9 per cent of 169 operated patients and 45·0 per cent of the overall 338 patients) had index admission cholecystectomy. Given that the severity of disease in the snapshot study was right‐skewed towards a lower grade of severity, it could be surmised that factors relating to local culture and logistics were predominant in the decision to pursue a non‐operative approach on index admission and to defer cholecystectomy.

When asked why index admission cholecystectomy was not performed more frequently in their centre, despite TG18 best practice, respondents cited lack of timely access to an emergency operating room, delayed patient presentation, local surgical culture, or surgeon preference for interval cholecystectomy. Although the effect of unscheduled admissions on hospital bed capacity and the knock‐on ability of a centre to deliver elective surgical care was beyond the scope of this study, it may reasonably be presumed that uncoupling elective and emergency service lines and resource allocation would facilitate and incentivize definitive care of index admissions. Indeed, respondents working in centres employing a resourced dedicated acute care surgery service line were significantly less likely to cite surgeon preference as the reason for poor rates of index admission cholecystectomy (*P* = 0·013).

Recognizing that prompt diagnosis of biliary sepsis and initiation of appropriate antimicrobial therapy and source control are cornerstones in the treatment of acute cholecystitis and biliary pancreatitis, TG18 recommends obtaining blood cultures on admission^14^. However, just 24 per cent of respondents reported following this guideline for cholecystitis and 32 per cent for pancreatitis. When practice was analysed in the snapshot audit, the proportions were 30·5 and 28·2 per cent for patients with cholecystitis and pancreatitis respectively. Instead, this step was omitted and empirical broad‐spectrum antibiotic therapy was commenced, predominantly using piperacillin–tazobactam (in 40·9 per cent of patients in the snapshot audit).

Self‐report adherence to TG18 recommendations among participating hospitals, coupled with snapshot audit data, shows low compliance in the domains of index admission surgical management and culture‐guided antimicrobial therapy in patients with complicated biliary disease. Centres that used an acute care surgery model were significantly more likely to perform laparoscopic cholecystectomy during the index admission of patients with complicated biliary calculous disease than general surgeons who predominantly performed elective procedures but provided an on‐call commitment. Individual recommendations of the TG18 each represent a marginal improvement towards homogenizing evidence‐based best surgical practice in the management of complicated biliary calculous disease. The design of the present non‐interventional, purely observational, study – aggregating the experience of many different university and community hospitals in different countries and healthcare systems, each with a different model for the provision of acute surgery – yields a representative insight into real‐world practice and, the authors hope, highlights challenges in achieving all of the TG18 recommendations. Recognition of these practice‐related cultural and logistic limitations, and perhaps movement towards an acute care surgery model of unscheduled surgical care, may allow closer alignment of guidelines and practice.

## Collaborators

Members of the ESTES Cohort Studies Group: A. Shamiyeh, L. Rosetti, G. Klimbacher, B. Klugsberger (Kepler University Clinic, Linz, Austria); P. Healy, C. Moriarty, C. Power, N. Knightly, A. D. K. Hill (Beaumont Hospital, Dublin, Ireland); D. C. Winter, M. E. Kelly, B. E. Creavin, É. J. Ryan, C. C. Duffy (SVUH Institute for Clinical Outcomes Research and Education, Dublin, Ireland); M. Sugrue, M. H. Moore, L. Flanagan (Letterkenny University Hospital, Letterkenny, Ireland); J. Ryan, C. Keady, B. Fahey, K. L. McKevitt, K. Barry (Mayo University Hospital, Castlebar, Mayo, Ireland); K. C. Conlon, K. Mentor, A. Kazemi‐Nava, B. Julies (Saint Vincent's University Hospital, Dublin, Ireland); P. F. Ridgway, D. O. Kavanagh, M. Whelan, M. Donnelly, C. McCarrick, U. Muhammad, T. M. Connolly, P. C. Neary (Tallaght University Hospital, Dublin, Ireland); S. Magalina, V. Cozza, A. LaGreca, D. Gui (Fondazione Policlinico Universitario ‘A. Gemelli’ IRCCS, Rome, Italy); A. Malagnino, M. Zago, M. Montuori (Policlinico San Pietro, Bergamo, Milan, Italy); A. Biloslavo, N. Samardzic, S. Fracon, D. Cosola, N. de Manzini (Clinica Chirurgica, Trieste University Hospital, Trieste, Italy); U. Fernandes, P. Avelar, R. Marques, A.S. Esteves, A. Marçal, C. Gomes (Centro Hospitalar de Trás‐os‐montes e Alto Douro, Vila Real, Portugal); D. Machado, T. Teles, S. Neves, M. Semiao, R. Cunha (Centro Hospitalar Cova de Biera, Covilha, Portugal); J. Pereira, J. Constantino, M. Sá, C. Casimiro (Centro Hospitalar Tondela‐Viseu, Viseu, Portugal); L. Ionescu, R. Livadariu, L. Stirbu, R. Danila, D. Timofte, B. Astefaniei (St. Spiridon Emergency Universitary Hospital, Iasi, Romania); A. Landaluce Olavarria, B. Estraviz Mateos, J. Gonzalez Taranco, D. Gomez, J. Barrutia, J. Zeballos (Hospital Urduliz, Bizkaia, Spain); D. Morales Garcia, A. Lozano Najera, E. Gonzalez Tolaretxipi (University Hospital ‘Marques de Valdecilla’, Santander, Spain); L. Tallon‐Aguilar, J. Pintor‐Tortolero, A. Sanchez‐Arteaga, V. Duran‐Muñóz Cruzado, V. Camacho‐Marente, J. Tinoco‐Gonzalez (Hospital Virgen del Rocío, Seville, Spain); A. Älverdal (Jönköping Medical Center, Jönköping, Sweden); S. Redeen (Linköping University Hospital, Linköping, Sweden); A. Mohammad, R. Ahl, M. Wikström (Örebro University Hospital, Örebro, Sweden); S. Marinos, N. Warner, R. Patel, T. Magro, R. Sunthareswaran (Stoke Mandeville Hospital, Aylesbury, UK); A. Mihailescu, G. Pokusewski, A. L. Bubuianu (Tameside General Hospital, Ashton‐under‐Lyme, UK); C. Dimitriu, M. Paraoan (Wrightington, Wigan and Leigh NHS Foundation Trust, Wigan, UK); A. Desai, K. Jones, M. Mlotshwa, K. Ross, S. Lambracos, Y. Tryliskyy (Worthing Hospital, Worthing, UK); D. C. Cullinane (Marshfield Clinic, Marshfield, Wisconsin, USA).

## Disclosure

The authors declare no conflict of interest.
